# Clinical Decision-Making Case: A Giant Headache

**DOI:** 10.21980/J8.52322

**Published:** 2025-12-31

**Authors:** Mark Portman, Linda Herman

**Affiliations:** * Sutter Roseville Medical Center, Department of Emergency Medicine, Roseville, CA

## Abstract

**Audience:**

This certifying exam practice case is intended for emergency medicine residents and medical students rotating through emergency medicine.

**Introduction:**

Giant cell arteritis (also known as GCA, temporal arteritis, cranial arteritis, or Horton’s disease) is the most common systemic vasculitis.^1^ Patients commonly present with a new and unique headache, often with tenderness in the temporal region. Patients may present with associated jaw claudication and transient visual loss. Constitutional symptoms such as fever and fatigue are common and proximal muscle weakness may be present with concurrent polymyalgia rheumatica.^1^ These symptoms are thought to be a result of an exaggerated immune response to vascular injury with lymphocyte proliferation and giant cell formation which can lead to luminal narrowing and even ischemia.^2^

The incidence varies among various demographics. It is highest in Scandinavia (21 per 100,000) and lowest in East Asia (less than one per 100,000). Globally, it is estimated to be 10 per 100,000 and in the US roughly 19 per 100,000.^2^,^3^ Recent studies have shown increasing incidence in Hispanic and African American populations while showing it to still be rare in Middle Eastern and Asian populations.^4^ It is most common in patients who are 70–79 years old and almost never presents in those under 50.^1^

Left untreated, GCA can result in significant morbidity – vision loss.^4^ Treatment is not benign – there are adverse effects of long-term glucocorticoid therapy. The cost to the healthcare system in the US is expected to increase dramatically by 2050 due to the aging population and cost of treatment.^3^

Given its relatively low incidence but high morbidity, giant cell arteritis is a rare diagnosis that Emergency Medicine residents may not encounter in training but is an important differential diagnosis to consider in the appropriate clinical context.

**Educational Objectives:**

By the end of this clinical decision-making case, learners will be able to: 1) demonstrate increased knowledge pertaining to ABEM’s clinical decision-making case, 2) communicate the differential diagnosis of a new acute onset headache in patients over the age of 50 and the importance of giant cell arteritis in that differential, 3) acquire an appropriate history and physical exam in this clinical setting, 4) verbalize, interpret, and justify the appropriate diagnostic testing for this clinical case (at minimum CT head, complete blood count (CBC), basic metabolic panel (BMP), comprehensive metabolic panel (CMP), erythrocyte sedimentation rate (ESR), and 5) explain the appropriate treatment and disposition of a patient with temporal arteritis.

**Educational Methods:**

This session was structured after the clinical decision-making case that was introduced by the American Board of Emergency Medicine (ABEM) in the instructional videos on the ABEM Qualifying Exam Part 2 released in December 2024. The materials used were modeled after the samples that were provided in the supplemental material for the clinical decision-making case. Slides were provided to the instructor concerning clinical presentation, differential diagnosis, and management for the debriefing following the session. This case was tested using 18 resident volunteers PGY 1–2 in an ACGME (Accreditation Council for Graduate Medical Education) accredited emergency medicine residency program.

**Research Methods:**

Using a score sheet, evaluators assessed the residents’ performance in acquiring appropriate clinical information, interpreting diagnostic tests, providing a differential, and justifying their management. Residents were asked to evaluate the educational value of the case.

**Results:**

Nine PGY1 residents and nine PGY2 residents completed the case, scoring 19.7/25 with four failures and 21.4/25 with three failures, respectively. Reasons for failing included scoring less than 19/25 or missing a critical action. Of the 17 residents that completed the post-survey, the educational value was reported to be 4.7/5 with all residents stating it increased their medical knowledge. Almost all of those residents stated that this experience made them more comfortable with the new ABEM clinical decision-making case.

**Discussion:**

This educational case focusing on giant cell arteritis (GCA) was effective in enhancing resident knowledge and clinical skills. All participating residents reported increased knowledge following the exercise and rated the case highly in terms of educational value. Performance outcomes further supported the case’s efficacy. All residents successfully completed critical actions related to obtaining an adequate history, performing a focused physical exam, and ordering appropriate diagnostic tests. The only resident who failed the critical action of starting steroids was also the only resident to miss the critical action of diagnosis, which supports the close association of these two critical actions. This positive feedback supports the use of this case to expose learners to a rare but high-risk diagnose which they are unlikely to encounter during clinical training.

**Topics:**

Giant cell arteritis, temporal arteritis, headache, vasculitis, ophthalmologic disorders.

## USER GUIDE

**Table:** 

List of Resources:	
Abstract	239
User Guide	241
For Examiner Only	243
Certifying Exam Assessment	250
Stimulus	252
Debriefing and Evaluation Pearls	259

**Learner Audience:**

Interns, Junior Residents, Senior Residents

**Time Required for Implementation:**

Case: Clinical Decision-Making cases are 15 minutes as directed by American Board of Emergency Medicine (ABEM).

Debriefing: 5 minutes

**Recommended number of learners per instructor:**

1–3

**Topics:**

Giant cell arteritis, temporal arteritis, headache, vasculitis, ophthalmologic disorders.

**Objectives:**

By the end of this clinical decision-making case, the resident/med student will:

1. Demonstrate increased knowledge pertaining to ABEM’s clinical decision-making case
2. Request an appropriate history and physical exam in this clinical setting
3. Communicate the differential diagnosis of a new acute onset headache in patients over the age of 50 and the importance of giant cell arteritis in that differential
4. Verbalize the appropriate diagnostic testing for this clinical case (at minimum CT head, CBC, CMP, ESR) and be able to justify the indication for those tests
5. Explain the appropriate treatment and disposition of a patient with temporal arteritis

### Linked objectives, methods and results

The clinical decision-making case we designed was developed to be nearly identical to the ABEM Certifying Exam. This was done by creating a parallel structure to the ABEM test format as seen in the ABEM certifying exam sample case video (objective 1). The goal was to give examinees practice for the certifying exam. In addition, this case evaluated learners’ ability to obtPain a sufficient history and physical in a patient presenting with clinical features of giant cell arteritis (objective 2) and then communicate an appropriate differential (objective 3). They then were assessed on their diagnostics, treatment and disposition, and asked to justify their decision-making (objectives 4 and 5). To ensure the learners would meet these objectives, at the end of the interview a 5-minute debrief period was conducted to go over key historical and physical exam components and critical actions of this case.

### Recommended pre-reading for instructor

- Long, B. Episode 76: Giant Cell Arteritis. emDOCs Podcast. Apr 25, 2023. Accessed November 21, 2024. https://www.emdocs.net/emdocs-podcast-episode-76-giant-cell-arteritis/.
- Ostermayer D, Lin P, Donaldson R, et al. Giant Cell Arteritis. WikiEM. Published Oct 11, 2023. Accessed Dec 3, 2024. https://wikem.org/wiki/Giant_cell_arteritis

### Results and tips for successful implementation

We made a schedule that allowed 18 residents to be tested by a faculty member. We scheduled this oral boards practice case during didactics and had three faculty proctoring three different oral boards cases. Every resident had the same faculty proctor for each case. Each case took 20 minutes, and for 18 residents, this took roughly 3.5 hours including breaks. Other faculty led small group case discussions and simulations during the downtime outside of the oral boards cases. We simplified our scoring method based on points and critical actions. Residents were required to score 19/25 points (76%) and not miss any critical actions to pass.

Residents were asked to evaluate the educational value of the case using a 1–5 Likert scale (5 being excellent). Evaluators were asked to score the residents by designating whether or not the learner was able to provide the correct responses concerning required appropriate historical information, physical examination characteristics, diagnostic testing needed, forming a correct differential, interpreting diagnostic results, and reaching the correct diagnosis, management, and disposition of the patient while providing correct transition-of-care information. The examiner would mark the evaluation form with either a yes or no.

Nine PGY1 residents and nine PGY2 residents, thus eighteen residents in total, completed the case. The PGY1 residents’ mean score was 19.7/25 points. Four of the nine participants failed - three due to a score less than 19/25, one for missing the critical action of calling a consultant. The PGY2 residents’ mean score was 21.4/25 points. None failed by scoring less than 19/25 points but three failed for missing the critical action of calling a consultant. Only one resident did not determine the correct diagnosis. The most frequent error was choosing the incorrect disposition which was admitting instead of discharging the patient with follow-up.

Seventeen residents completed the post-survey. The learners rated the educational value of the case 4.7/5. Ten residents stated that the case increased their knowledge “somewhat” about the disease process while seven stated that it definitely increased their medical knowledge. Fifteen residents said that the experience made them more comfortable with the new process but that they needed more practice. One resident stated that they were very comfortable with the process, and one other stated that they were somewhat uncomfortable but knew how to study for the new testing procedure.

PGY-2 residents appropriately scored better than their PGY-1 counterparts. PGY-2s had a higher mean score (21.4/25, 86%) and all passed based on our predetermined threshold (19/25, 76%). Although three PGY-2s did not contact a consultant, they still arrived at the correct diagnosis, initiated appropriate treatment, and determined correct disposition with appropriate follow up. This raises the question of whether consultant contact, while important, was necessarily a critical action.

PGY-1 residents had a lower mean score (19.7/25, 79%) and a higher failure rate, with four residents not passing—three by score and one for omitting the critical action of calling a consultant.

The most frequent non-critical error across both groups involved incorrect disposition. Twelve out of eighteen residents initially opted for admission instead of discharge. Encouragingly, nine of those twelve still contacted a consultant, a step that may have prompted reconsideration of their management plan in a real clinical scenario. One important takeaway was the benefit of simplifying the scoring system. Our original rubric used a 37-point scale with variable weights for different actions. This proved cumbersome and difficult to apply consistently. Transitioning to a 25-point system improved scoring clarity without compromising the ability to appropriately differentiate performance.

We selected multiple critical actions that seemed crucial to providing appropriate care to both a patient with an undifferentiated, atypical headache, and to GCA. These included minimum standards for history-taking, physical examination, and diagnostic testing. While CT imaging is not required in the diagnosis of GCA, it was considered a critical action in this case, given that the patient was elderly, presented with an atypical headache, and had a neurologic deficit. Accurate diagnosis, initiation of steroids, and contacting a consultant were also deemed essential actions, given their direct impact on patient outcomes.

In summary, this simulation case was well-received by residents and demonstrated value in teaching the recognition and management of GCA. It not only provided exposure to a rare diagnosis but also tested residents clinical approach to undifferentiated headaches. Simplifying the scoring system proved beneficial and will inform future simulation development. We plan to continue using this case for oral board preparation and as a high-yield educational tool for emergency medicine residents.

## Figures and Tables

**Table:** 

**I. History**		Yes	No
**1a**	Asked about acuity, onset (rapid vs. gradual), fall/trauma, focal neurologic deficit, fevers, medications/recent changes?		
**1b**	Asked about above and the location of pain, prior migraine history?		
**2a**	Medications: Asks about medications eliciting risk of ICH (anticoagulation) or possibility of rebound headache if recently stopped a medication?		
**2b**	Onset: Explains timing of onset allows to risk stratify for subarachnoid hemorrhage or dissection with severe, rapid onset “thunderclap” headaches.		
**II. Physical Examination**			
**3a**	Asked about: neurologic exam, HEENT		
**3b**	Asked about above and general appearance, palpates temporal arteries.		
**4**	Neuro exam: Explains focal neurologic deficits suggesting emergent pathologies.		
**5**	Tenderness of temporal arteries suggests GCA.		
**III. Differential Diagnosis**			
**6a**	Giant cell arteritis.		
**6b**	Gives two other critical diagnoses (tension headache, migraine headache, intracranial mass, intracranial hemorrhage, meningitis).		
**7**	Explains historical and physical findings concerning for GCA and the importance of early intervention to avoid vision loss.		
**8**	Intracerebral hemorrhage is a can’t miss diagnosis, and her age and general weakness increases the concern?		
**IV. Diagnostic Studies**			
**9a**	CBC, CMP, CT head		
**9b**	Inflammatory markers.		
**10**	Head CT to rule out mass and hemorrhage.		
**11**	Elevated inflammatory markers are associated with GCA.		
**V. Treatment and Other Actions**			
**12a**	Started on steroids.		
**12b**	Called consultant.		
**13**	Steroids decrease risk of vision loss and should be started immediately?		
**14**	Consultation for close follow-up and temporal biopsy within one week.		
**VI. Final Diagnosis**			
**15**	Temporal arteritis		
**VII. Disposition**			
**16**	Discharge the patient?		
**17**	Answer either “visual deficits” or “intractable pain” or both?		
**VIII. Transitions of Care**			
**18a**	Prescribe steroids, close outpatient referral, give return precautions (severe pain, vision changes)?		
**18b**	Coordinate referral for biopsy?		

**Table:** 

**Candidate Task Sheet**	
**#1**	**Emergency Department Admitting Form**
**#2**	**Historical Information**
**#3**	**Physical Exam Findings**
**#4**	**Diagnostic Studies: Laboratory Results**
**#5**	**Head CT**

**Table:** Clinical Decision-Making Task Sheet

**CASE PARAMETERS**
This is a 15-minute case You will interact with two examiners. This is an interview style without role playing; you should simply reply to the questions asked. You may be interrupted to move you through the case; this is not a reflection of your performance.
**PATIENT INFORMATION**
Patient had a gradual onset of frontal headache about 24 hours ago.
**VITAL SIGNS**
BP: 132/72 P: 74 R: 16 T: 100.2° F 02Sat: 100% on RA
**TASK STATEMENT**
Your tasks are as follows: List pertinent elements of a focused history and physical exam Develop an appropriate differential and/or provisional diagnosis Select and interpret appropriate studies Articulate appropriate patient management including discharge instructions

**STIMULUS 1:** Emergency Department Admitting Form

**Patient Information**	
**Patient Name**	Gloria Jones
**Age**	72
**Gender**	Female
**Method of Arrival**	Private auto
**Chief Complaint**	Frontal headache
**Vital Signs on ED Arrival**	BP: 132/72 P: 74 R: 16 T: 100.2*°* F O2 sat: 100% Weight: 70kg

**STIMULUS 2:** Historical Information

**History of Present Illness/Description of Event**	
The patient developed nonspecific aches and pains three weeks ago and has noted pain in her jaw with chewing. Soreness seems worst in the mornings. She has also noticed some difficulty rising after being seated for any length of time. Naproxen provides some relief. Frontal headache started 24 hours ago with soreness over her frontal scalp, especially in the temporal areas. She describes the pain as aching in the frontal region from temporal area to temporal area Denies vomiting, trauma, focal neurological deficits, or change in vision.	
**History**	
**Past Medical History**	Denies
**Past Surgical History**	Tonsillectomy as a child, hysterectomy 10 years ago for excessive bleeding
**Medications**	Denies
**Allergies**	No known drug allergies
**Social**	Non-smoker, social alcohol use

**STIMULUS 3:** Physical Exam Findings

**Physical Examination**	
**General Appearance**	Well-developed, well-nourished woman who appears younger than her stated age, uncomfortable but cooperative.
**Head**	Normocephalic, atraumatic, tenderness over the temporal arteries bilaterally, temporal artery pulses appear weak to palpation.
**Eyes**	Pupils equal, react to light, extraocular muscles intact, sclera anicteric, conjunctiva pink, fundi clear with sharp disks, normal visual acuity.
**Ears**	Tympanic membranes clear bilaterally.
**Oral**	Normal oropharynx, if happens to chew should complain of pain in cheeks after about 5 – 10 minutes.
**Neck**	Supple, nontender, no jugular vein distention, no lymphadenopathy, no thyroidomegaly, trachea midline.
**Neurologic**	Cranial nerves intact, no pronator drift, good coordination, strength appears to be normal though patient appears to be in some pain when standing up for portions of the exam, reflexes two plus and symmetrical, sensation normal.
All other components of the physical exam are within normal limits	

**STIMULUS 4 t8-jetem-10-5-ce239:** Diagnostic Studies and Results

**CBC**	WBC	9.8/mm^3^	(Normal 4.0 – 11.0 × 1000/mm^3^)
	Hgb	11.0g/dL	(Normal 11.7 – 15.5 g/dL)
	Hct	33%	(Normal 35.0 – 47.0%)
	Platelets	152,000/mm^3^	(Normal 150 – 400 × 1000/mm^3^)
	Differential		
	Segs	66%	(Normal 50 – 70%)
	Bands	2%	(Normal 0 – 6%)
	Lymphs	31%	(Normal 25 – 35%)
	Monos	1%	(Normal 4 – 6%)
	Eos	2%	(Normal 1 – 3%)

**CMP**	Na^+^	136mEq/L	(Normal 136 – 145 mmoL/L)
	K^+^	3.8mEq/L	(Normal 3.5 – 5.1 mmol/L)
	CO_2_	24mEq/L	(Normal 21 – 32 mmol/L)
	Cl^−^	91mEq/L	(Normal 98 – 107 mmol/L)
	Glucose	112mg/dL	(Normal 70 – 100 mg/dL)
	BUN	21mg/dL	(Normal 6–25 mg/dL)
	Creatinine	1.2mg/dL	(Normal 0.40 – 1.00 mg/dL)

**ESR**	88 mm/hour		(Normal 0–20 mm/hour)

**CRP**	38 mg/L		(Normal 0–3 mg/L)

**STIMULUS 5 f1-jetem-10-5-ce239:**
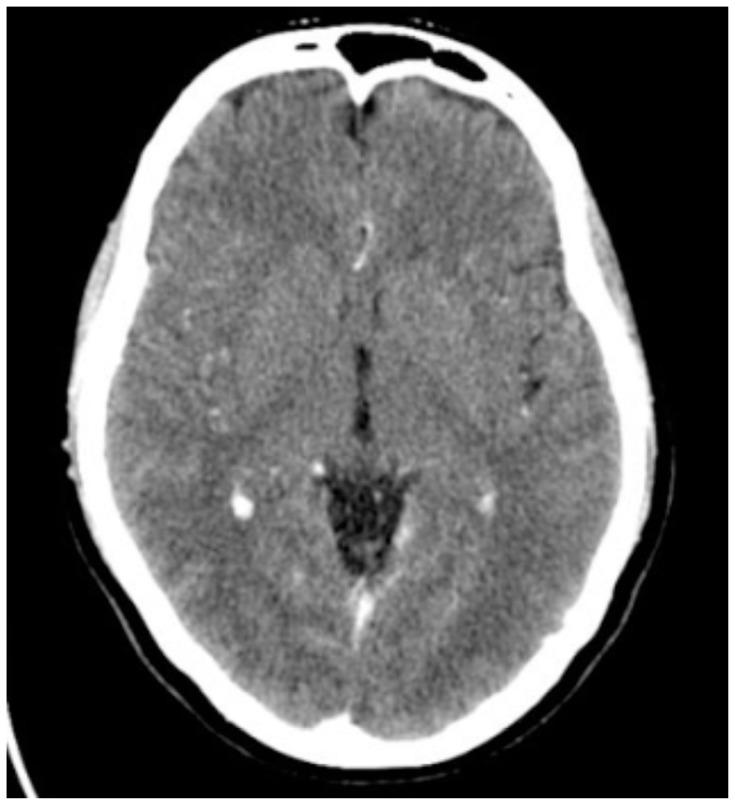
CT Head^8^

## FOR EXAMINER ONLY

### Clinical Decision-Making Case: A Giant Headache Summary

**Diagnosis:** Giant cell arteritis (temporal arteritis)

**Case Summary:** This case involves a 72-year-old female who presents with complaints of a bitemporal headache and multiple myalgias. The woman began to have nonspecific myalgias three weeks prior to admission and noticed that she was having difficulty rising from bed and chairs but her primary doctor thought it was just due to age. She has noted stiff shoulders and hips, worse in the morning and reports having intermittently run a low-grade fever. She began to have a new and unusual headache approximately 24 hours ago which is unrelieved with Naproxen or acetaminophen.

She has no past medical history. Her only surgical history is a tonsillectomy as a child and vaginal hysterectomy ten years ago for vaginal bleeding without endometrial or cervical cancer. She has no allergies or any pertinent social history.

The candidate must obtain history consistent with polymyalgia rheumatica, discover the tenderness over the temporal arteries, order an erythrocyte sedimentation rate, start steroids orally and consult a neurologist (or another appropriate consultant) regarding follow up and obtaining a temporal artery biopsy.

### Clinical Decision-Making Case: A Giant Headache Examiner Script

**Case Introduction:**

“Hello Doctor, this is a clinical decision-making case. There is no role playing. In response to the questions I will ask, please give me a LIST of information you would gather to come to a final diagnosis. At times, I may interrupt you to move you through the case; this is not a reflection of your performance. You will have 15 minutes to complete the case. Before we begin, do you have any questions?”

“The patient we will be discussing is a 72-year-old female who presents to the emergency department with a frontal headache. Her initial information is here on the screen.”

Provide Learner Stimulus #1

#### HISTORY

Prompt 1:

What additional historical information would you want to know?

Scoring Guidelines:

The 10 key components for the history are: acuity of headache, whether acute/new vs chronic; onset of headache, rapid vs gradual; location of pain; history of fall/trauma; vision changes; focal weakness or numbness; associated fevers; associated meningismus (either neck pain/stiffness or photophobia); prior history of migraines, (is this one similar?); medications/recent changes

Rationale: The above is based on a recommended general approach to obtaining clinical information for headaches. The examinee should get at least 6/10 historical elements and 8/10 to score higher.

*If the learner lists less than 6/10 key history components, prompt them:*

“Are there any additional historical components that would be helpful?”

Prompt 2a:

“Why would it be important to ask the patient about medications and/or medication changes?”

Scoring Guidelines:

Rationale: Learner should include an explanation regarding blood thinners and risk for intracranial hemorrhage (ICH) or stopping medications and having rebound headache symptoms.

Prompt 2a:

“Why is the timing of onset of symptoms (rapid vs gradual) important to know in a patient with this presentation?”

Scoring Guidelines:

Rationale: Learner should explain the concern for subarachnoid hemorrhage (SAH), sentinel hemorrhage/herald bleed, or carotid/vertebrobasilar artery dissection with severe, rapid onset “thunderclap” headaches.

#### PHYSICAL EXAMINATION

“Thank you, doctor, here is the additional historical information.”

Provide Learner Stimulus #2 and read pertinent information.

Prompt 3:

“Now would you please discuss what you will be assessing on the physical exam of this patient.”

Note for examiner: if any exam stated is not specific enough, you can ask the learner to “clarify their x exam.”

Scoring Guidelines:

Rationale: General appearance; HEENT (neck stiffness/range of motion, masses/abrasions/asymmetry, basic eye exam, congestion/rhinorrhea, ear exam/mastoid process); neurologic exam (cranial nerves, weakness, paresthesias); palpate temporal arteries

Prompt 4:

“Why is a neurologic exam important in a patient who presents with headache?”

Scoring Guidelines:

Rationale: Learner should include an explanation regarding focal neurological deficits being associated with concerning pathology such as intracranial masses, an arterial dissection, hemorrhagic stroke, venous sinus thrombosis.

Prompt 5:

“Why would palpation of the temporal arteries and scalp be important in a patient with this presentation?

Scoring Guidelines:

Rationale: Learner should include an explanation that this can be associated with giant cell arteritis.

#### DIFFERENTIAL DIAGNOSIS

“Thank you, doctor, here are the physical exam findings.”

Provide Learner Stimulus #3 and read pertinent information.

Prompt 6:

“Based on what you now know, what are the top three items on your differential diagnosis based on the most likely conditions?” (If more than three conditions are mentioned, say, “OK, thank you. Please give me your three, and only three, most likely diagnoses.”)

Scoring Guidelines:

Rationale: The leaner should answer some combination of: g*iant cell arteritis (temporal arteritis), meningitis, intracranial hemorrhage, intracranial mass, tension headache, migraine headache*

Prompt 7:

“Given this patients presentation and physical exam findings, why would it be important to consider giant cell arteritis (temporal arteritis) in the differential?”

Scoring Guidelines:

Rationale: The learner should include an explanation that this patient has many appropriate historical and physical exam elements: age, biological sex, associated generalized weakness, location of headache, tenderness in the temporal region, and the importance of diagnosis for early intervention to prevent vision loss.

Prompt 8:

“Why would it be important to consider intracranial hemorrhage (ICH) in a patient with this presentation?”

Scoring Guidelines:

Rationale: The learner should include an explanation that this patient is elderly, it is an atypical headache for her, and she has weakness on her physical exam. They may also explain it is relatively common among the rare, dangerous pathologies causing headaches and that it should not be missed.

#### DIAGNOSTIC STUDIES

Prompt 9:

“Based on what you know and your working differential diagnosis, what, if any, diagnostic studies would you order?”

Scoring Guidelines:

Rationale: Complete blood count (CBC), Basic metabolic panel (BMP), Liver function tests (LFTs), Erythrocyte sedimentation rate (ESR), C-reactive protein (CRP), head CT

Prompt 10:

“Doctor, in a patient with this history why would ordering a head CT be important?”

Scoring Guidelines:

Rationale: The learner should include an explanation that a CT can evaluate for an intracranial hemorrhage and mass. This patient’s atypical headache, age, and duration of symptoms can all justify this test.

Prompt 11:

“Doctor, in a patient with this history and exam findings, why would inflammatory markers be important to order?”

Scoring Guidelines:

Rationale: The learner should include an explanation that these can be associated with GCA.

Provide Learner Stimuli #4–5

#### TREATMENT AND OTHER ACTIONS

Prompt 12:

“Based on what you now know, which treatments, if any, would you order and/or what actions, if any, would you perform?”

Scoring Guidelines:

Rationale: The learner should request to start the patient on steroids and call a consultant (neurologist, ophthalmologist, or rheumatologist should be acceptable).

Prompt 13:

“Doctor, in a patient presenting with possible giant cell arteritis, steroids are an essential treatment. Why and how soon should those be given?”

Scoring Guidelines:

Rationale: The learner should include an explanation that steroids decrease risk of vision loss. They may also explain that it is OK to start immediately and will not interfere with biopsy if performed within seven days.

Prompt 14:

“Doctor, in a patient with possible giant cell arteritis, consultation with a specialist is important; why is it important and what specific follow-up tests are needed?”

Scoring Guidelines:

Rationale: The learner should include an explanation regarding the patient needing close follow up in 24–48 hours and a temporal artery biopsy within a week.

#### FINAL DIAGNOSIS

Prompt 15:

“Based on everything you know about this case, what is your final diagnosis?”

Scoring Guidelines:

Rationale: Verbalizing giant cell arteritis or temporal arteritis meets the critical action.

#### DISPOSITION

Prompt 16:

“Based on what you know, what should be the disposition of this patient?”

Scoring Guidelines:

Rationale: Discharge

Prompt 17:

“With the same diagnosis, in what situation might you consider admitting this patient?”

Scoring Guidelines:

Rationale: Consider admission for visual deficits, intractable pain.

#### TRANSITION OF CARE

Prompt 18:

“What specific actions would you take at the time of discharge? This may include discharge instructions, prescriptions, return precautions.”

Scoring Guidelines:

Appropriate specific actions include a start on steroid prescription, close outpatient follow up with specialist, referral for temporal artery biopsy, return precautions (severe pain, vision changes), steroid precautions (GI bleed risk, hyperglycemia, mania/psychoses).

Rationale: Ensuring outpatient follow up and temporal artery biopsy are important for ongoing care. Specific return precautions and a discussion about glucocorticoid therapy are also important components to providing adequate discharge instructions.

*Thank you, Doctor. That concludes this case.*

*Please tear up your notes.*

## CERTIFYING EXAM ASSESSMENT

*Clinical Decision-Making Case: A Giant Headache*

Learner: _________________________________________

**Summative and formative comments:**

### Stimulus Inventory

[Table t8-jetem-10-5-ce239]

[Fig f1-jetem-10-5-ce239]

## DEBRIEFING AND EVALUATION PEARLS

### Clinical Decision-Making Case: A Giant Headache

- When assessing a patient with an atypical headache, always ask about acute vs chronic, rapid vs gradual onset with headaches. Always ask about medications and any recent changes. Also, elicit information regarding falls and perform an exam assessing for trauma and neurologic deficits (including vision changes).^5^
- Risk factors include biological sex female, age 70–79.^1^
- Starting steroids can help prevent the dreaded complication of vision loss. Early discussion with a consultant to help guide treatment and arrange follow up is crucial.^6^, ^7^
- Patients may present with a temporal headache, jaw claudication, concurrent polymyalgia rheumatica (with proximal muscle weakness).^7^
- Left untreated, it can result in vision loss.^7^
- Diagnosis is initially clinical, supported by elevated inflammatory markers, and confirmed with temporal artery biopsy.^6^
- Treatment is long term glucocorticoid therapy and requires outpatient follow up with multiple specialists, often including rheumatology, neurology, and ophthalmology.^4^
